# Sacrificial CaCO_3_ Precoating Preserves Fouling Reversibility of Ceramic Nanofiltration During Filtration of Real Wastewater Treatment Plant Effluent

**DOI:** 10.3390/membranes16090306

**Published:** 2026-09-18

**Authors:** Yuke Li, Luuk C. Rietveld, Sebastiaan G. J. Heijman

**Affiliations:** Section of Sanitary Engineering, Department of Water Management, Faculty of Civil Engineering and Geosciences, Delft University of Technology, Stevinweg 1, 2628 CN Delft, The Netherlands

**Keywords:** ceramic nanofiltration, sacrificial precoating, fouling reversibility, calcium carbonate, WWTP effluent, membrane cleaning

## Abstract

Fouling of ceramic nanofiltration (NF) membranes becomes difficult to reverse when complex wastewater constituents accumulate directly on the selective layer. Sacrificial precoating may spatially separate foulant deposition from the membrane surface, but its effectiveness under real wastewater conditions remains insufficiently understood. A single CaCO_3_ precoating step was evaluated over three consecutive filtration–cleaning cycles using municipal wastewater treatment plant (WWTP) effluent. During three consecutive 6-h filtration cycles, precoating substantially improved the reversibility of hydraulic fouling. Permeability recovery reached 92% after the first cleaning and was 80% after the second cleaning, whereas the uncoated membrane recovered only 34% after the first cleaning and subsequently showed little effective recovery. The precoated system also exhibited markedly lower irreversible hydraulic resistance. In addition to fouling control, the mineral precoat altered solute removal in the complex wastewater matrix: DOC removal remained approximately 90%, while apparent phosphate removal increased from 38% for the uncoated membrane to 90% for the precoated system. These findings demonstrate that CaCO_3_ precoating acts as a sacrificial interfacial layer that preserves fouling reversibility during ceramic NF treatment of real WWTP effluent, while mineral–solute interactions may additionally contribute to the apparent removal of selected inorganic solutes.

## 1. Introduction

Global water scarcity has intensified the need for advanced wastewater treatment technologies [1,2,3]. Nanofiltration (NF) is increasingly considered as a complementary process to conventional water treatment because of its ability to remove dissolved contaminants while maintaining relatively low chemical requirements [4,5,6,7,8]. Ceramic NF membranes are particularly attractive for municipal and industrial wastewater treatment because of their high chemical and mechanical stability [9,10,11,12,13]. Their nanoporous selective layers enable size- and charge-dependent separation of multivalent ions and low-molecular-weight compounds while maintaining high water permeability [14]. Despite these advantages, membrane fouling remains a major operational limitation [15,16]. During wastewater filtration, particulate matter, dissolved organic matter, and inorganic species can accumulate through pore blocking and surface deposition [17,18]. Interactions between organic macromolecules and colloidal or inorganic constituents may further promote the formation of compact or gel-like fouling layers [19]. More importantly, repeated filtration and cleaning may progressively convert readily removable surface deposits into persistent hydraulic resistance. Therefore, maintaining fouling reversibility is important for the sustained operation of ceramic NF membranes.

Current antifouling approaches include surface modification, incorporation of functional materials, and membrane precoating [20,21]. Among these approaches, precoating provides a removable intermediate layer between the feed water and the membrane surface. A sacrificial precoat formed by controlled deposition of particulate materials can intercept foulants before they directly contact the selective membrane layer, thereby reducing pore blockage and facilitating subsequent removal of the deposited layer [22]. Conventional precoating is generally achieved by circulating or filtering a particle suspension through the membrane module, followed by removal of excess particles [23]. Coagulant-assisted precoating, for example, has been applied to ceramic MF and UF membranes using iron- or aluminum-based materials to enhance natural organic matter removal through flocculation and entrapment [24]. Application to ceramic NF is more challenging because high-pressure backwashing may impose mechanical stress on module sealing interfaces, whereas low-pressure backwashing may be insufficient to remove strongly attached foulants [25]. Chemical cleaning is therefore commonly adopted as an essential supplementary measure for ceramic NF; however, repeated exposure to cleaning agents may compromise membrane structural integrity and long-term separation performance [19,26]. An alternative approach is to place a removable mineral layer on the membrane and direct foulant accumulation toward this sacrificial interface rather than the selective layer itself. Previous studies by Kramer et al. and Li et al. [25,27,28] investigated CaCO_3_ precoating combined with acid-assisted cleaning for ceramic NF, showing that dissolution of the mineral layer can promote detachment of surface-associated foulants. Quantitative improvements in permeability recovery have been reported under controlled water matrices. For example, Li et al. [27] achieved recoveries of 93% and 68% following 1st and 2nd cleaning cycles, respectively, using a fine-particle CaCO_3_ precoat. In a subsequent study using surface water, permeability recovery reached 95% and 87% following 1st and 2nd cleaning cycles for the precoated membrane, compared with only 39% and 2% for the uncoated membrane [28]. These results demonstrate the potential of CaCO_3_ precoating to preserve membrane cleanability during repeated operation. However, real wastewater treatment plant (WWTP) effluent contains a substantially more complex mixture of dissolved organic matter, colloids, multivalent ions, and nutrients. These constituents may alter both the development of the fouling layer and the interactions between the CaCO_3_ precoat and the wastewater matrix. It therefore remains unclear whether the improved fouling reversibility observed in simplified systems can be maintained under real wastewater conditions.

In the present study, CaCO_3_-precoated and uncoated ceramic NF membranes were compared during repeated filtration–cleaning cycles using real WWTP effluent. The study focused on permeability decline and recovery, the development of irreversible hydraulic resistance, and changes in organic and inorganic solute removal. Particular attention was given to whether CaCO_3_ precoating shifts fouling from persistent membrane-associated resistance toward a more removable interfacial deposit.

## 2. Materials and Methods

### 2.1. Membrane Structural and Performance Attributes

Commercial ceramic NF membranes (Inopor GmbH, Veilsdorf, Germany) consisted of an α-Al_2_O_3_ support, a γ-Al_2_O_3_ intermediate layer, and a mesoporous TiO_2_ selective layer (approximately 2–3 μm thick) [29]. The monolithic membranes contained 19 feed channels with an internal diameter of 3.5 mm. Each membrane was 0.5 m long and had an effective filtration area of 0.1045 m^2^ [30]. The ceramic NF membrane used in this study had an experimentally determined molecular weight cut-off (MWCO) of 1021 Da, based on polyethylene glycol (PEG) standards. PEG concentrations were analyzed by high-performance size-exclusion chromatography using a Shimadzu Prominence system equipped with a PSS GmbH column and a Shimadzu RID-20A refractive index detector [3]. Representative cross-sectional SEM images of the pristine ceramic membrane and the CaCO_3_-precoated membrane obtained using the same precoating procedure in our previous study are provided in Figure S1 [28]. The images confirm the multilayer ceramic membrane structure and visually demonstrate the formation of an additional CaCO_3_ layer on the TiO_2_ selective surface after precoating.

### 2.2. CaCO_3_ Nanoparticles for Membrane Precoating

In this study, the influence of CaCO_3_ particle precoat on ceramic NF membrane fouling was evaluated by employing laboratory-synthesized nanoparticles. The CaCO_3_ particles were prepared via aqueous-phase precipitation, where a calcium chloride (CaCl_2_·2H_2_O, ACS reagent grade) solution was titrated into sodium bicarbonate (NaHCO_3_, ≥99.7%) under controlled hydrodynamic conditions (stirring at 25 ± 0.5 °C, pH 8.5 maintained) [27]. Colloidal stabilization was engineered through surface charge modification, achieved by introducing 9 mM Ca^2+^ ions (as CaCl_2_·2H_2_O) into the suspension matrix to obtain a zeta potential of approximately +25 ± 0.5 mV, while bicarbonate alkalinity was regulated at 3 mM using a NaHCO_3_ buffer system. The particle size distribution (PSD) of CaCO_3_ was determined using a particle size analyzer (Microtrac Bluewave) with light scattering technology, followed by zeta potential measurements via Zetasizer (Malvern Instruments, Malvern, UK).

### 2.3. Feed Water Characteristics and Composition

WWTP effluent was collected from the Harnaschpolder WWTP, located in Den Hoorn, the Netherlands, with a treatment capacity of approximately 1.3 million population equivalents [31]. The effect of CaCO_3_ precoating on water-quality performance was evaluated by comparing the WWTP effluent with permeates obtained from the uncoated and precoated membrane systems.

DOC quantification was conducted using Shimadzu’s TOC-VPH analyzer employing standardized oxidative combustion-infrared detection methodology [32]. Ionic constituents, including inorganic species, were resolved and quantified via Metrohm ion chromatography (IC) employing stationary phases packed with ion-exchange resins, where separation occurs through differential ionic affinities [33]. All chromatographic analyses were performed under computer control using MagIC Net data acquisition and processing software. Feed and permeate samples were collected separately from each of three independent filtration experiments (*n* = 3) under the corresponding filtration conditions and were analyzed independently. The water-quality values reported in the main text represent mean values, while the corresponding standard deviations are provided in Table S1.

### 2.4. Configuration of Experimental Membrane Filtration System

**Figure 1:**
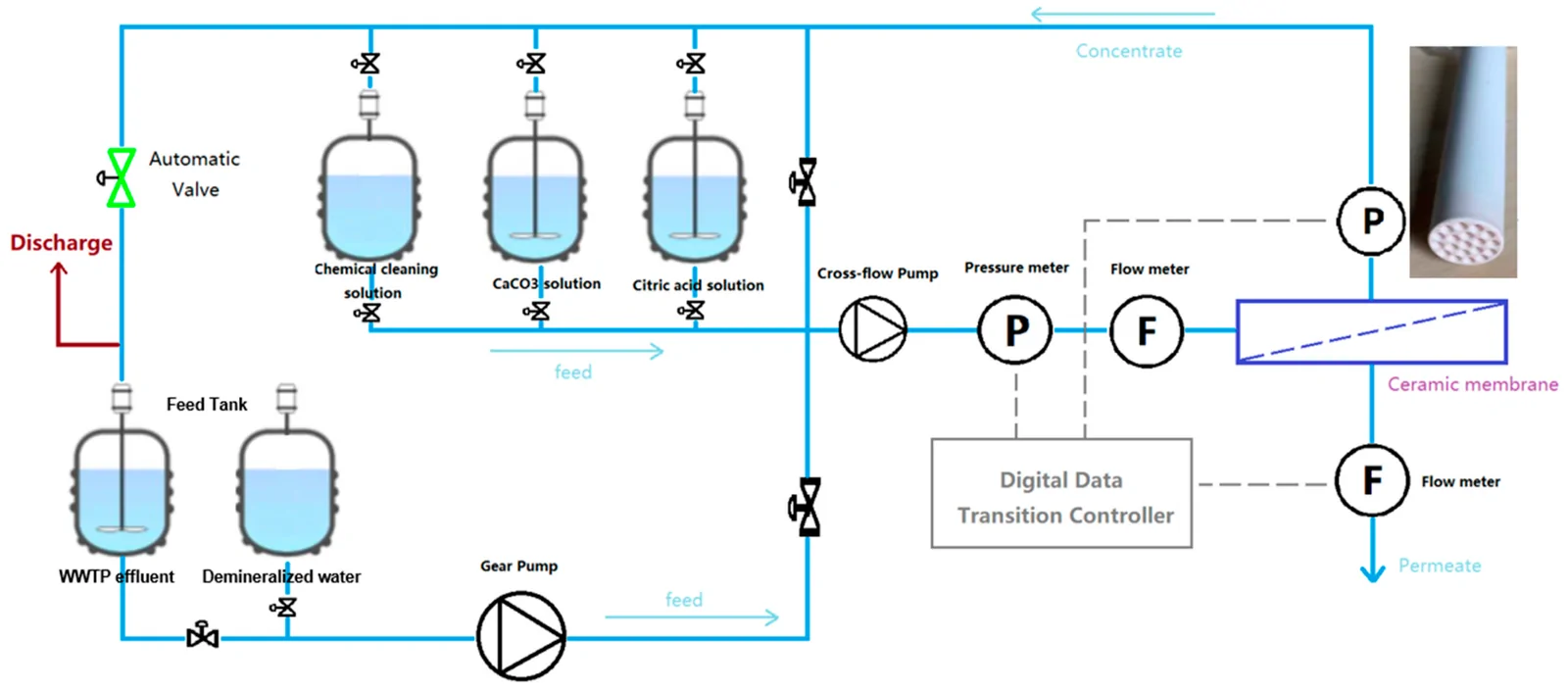
Bench-scale installation of cross-flow ceramic NF with constant flux.

A membrane-based cross-flow filtration setup was designed to operate under regulated hydrodynamic conditions, incorporating a dual-pump system that combined a feed pressure pump with a cross-flow circulation pump to ensure stable permeate production flows and consistent cross-flow speeds during all testing procedures (Figure 1). The study involved processing five different solutions in successive stages: (i) demineralized water to establish the baseline membrane performance and for rinsing after acid treatment; (ii) a CaCO_3_ suspension to form the initial precoat layer; (iii) WWTP effluent as a representative fouling solution; (iv) a 20 mmol L^−1^ citric acid solution for membrane regeneration between filtration cycles; and (v) a sodium hypochlorite solution for thorough oxidative cleaning between different experiments. The experimental procedure involved the formation of a CaCO_3_ precoat through dead-end filtration of a 0.4 g L^−1^ colloidal suspension. Precoating was performed at a constant flux of 120 L m^−2^ h^−1^ for 10 min, with the transmembrane pressure (TMP) maintained at approximately 10 bar. During WWTP-effluent filtration, a target permeate flux of 40 L m^−2^ h^−1^ was imposed, while the cross-flow velocity of 0.69 m s^−1^ (Re = 2415) was maintained [34,35]. The TMP was adjusted between 1 and 10 bar as required to maintain the target flux as fouling developed [27]. With the exception of demineralized water, all test solutions (both concentrate and permeate) were continuously cycled through the system’s feed tank (Figure 1) to preserve uniform solution properties during each experiment.

To facilitate the assessment of the membrane performance during the fouling experiment, membrane permeability was corrected for temperature-dependent changes in water viscosity using Equation (1) [36,37].

(1) $$L_{20{}^{\circ}C}=\frac{J\cdot e^{-0.0239\;\cdot \;\left(T-20\right)}}{∆P}$$

where *L* indicates the thermally adjusted membrane permeability with units of L·m^−2^·h^−1^·bar^−1^, *T* refers to the recorded aqueous solution temperature in degrees Celsius (°C), *J* represents the permeate flux (L·m^−2^·h^−1^), while Δ*P* stands for the pressure differential across the membrane.

To assess the recovery characteristics of membrane permeability, the normalized permeability ratio (*L*_1_*/L*_0_) was determined, where *L*_1_ (L·m^−2^·h^−1^·bar^−1^) represents the permeability under the present conditions, and *L*_0_ denotes the initial membrane permeability measured at the beginning of the experimental procedure. The study involved three key permeability assessments: (i) the determination of clean membrane properties before any fouling tests were performed; (ii) the analysis of permeability reduction following CaCO_3_ precoating; and (iii) the measurement of restored permeability using demineralized water after fouling and cleaning cycles to evaluate cleaning effectiveness. All filtration–cleaning experiments were independently repeated three times (*n* = 3). Inter-run variation in the principal hydraulic parameters was below 5%.

The extent of membrane fouling was evaluated by computing the fouling resistance as defined in Equation (2) [38,39,40]:

(2) $$J_{m}=\frac{△P_{m}}{\mu R_{t}}=\frac{△P_{m}}{\mu (R_{i}+R_{r}+R_{ir})}$$

where *J_m_* represents the permeate flux through the membrane (m s^−1^), Δ*P_m_* denotes the applied pressure differential across the membrane (Pa), and *μ* is the dynamic viscosity of the permeate (Pa·s). The resistance terms include *R_t_* for total system resistance, *R_i_* for the inherent membrane resistance, *R_r_* for reversible fouling components, removable by regular cleaning, and *R_ir_* for irreversible fouling that withstands regular cleaning procedures.

### 2.5. Chemical Cleaning

After each 6-h filtration cycle, both the CaCO_3_-precoated and uncoated membranes were cleaned with a 20 mmol L^−1^ citric acid solution for 15 min. The same citric acid cleaning protocol was applied to both systems to allow direct comparison of permeability recovery and fouling reversibility. After acid cleaning, the membrane system was rinsed with demineralized water before the subsequent filtration cycle. The CaCO_3_ precoat was applied only once before Cycle I and was not replenished before Cycles II or III. Three consecutive filtration–cleaning cycles were selected to evaluate whether the effect of a single precoating step could persist beyond the first filtration and cleaning event. This design enabled assessment of two successive cleaning events and the subsequent third filtration cycle, allowing the persistence of fouling reversibility to be evaluated under repeated operation. The three-cycle test was intended as a controlled short-term evaluation rather than as a simulation of long-term full-scale operation. After completion of each set of repeated filtration–cleaning experiments, a more intensive cleaning procedure was applied to restore the membrane to its initial condition before the next experimental series. Sodium hypochlorite (0.2%) was applied for 1 h to remove residual organic deposits [41]. For the CaCO_3_-precoated membrane, an additional treatment with 0.01 mol L^−1^ HCl for 5 min was used to dissolve remaining CaCO_3_ deposits [42]. The membrane was subsequently rinsed with demineralized water until the baseline permeability was restored.

## 3. Results and Discussion

### 3.1. Fouling Development and Reversibility During Filtration of WWTP Effluent

A CaCO_3_ precoat was deposited via dead-end filtration of a 0.4 g L^−1^ CaCO_3_ suspension at 10 bar and a permeate flux of 120 L m^−2^ h^−1^ for 10 min. Approximately 800 mg of CaCO_3_, corresponding to 7.7 g m^−2^ of membrane area, was deposited during precoating. The initial membrane permeability decreased from 16.8 to 15.6 L m^−2^ h^−1^ bar^−1^ after precoating, corresponding to a reduction of approximately 7%. The hydraulic penalty associated with formation of the sacrificial layer was therefore relatively small. The hydraulic stability of the CaCO_3_ precoat under cross-flow conditions has been demonstrated previously under comparable operating conditions [28]. In the present study, a similar cross-flow velocity of 0.69 m s^−1^ (Re = 2415) was employed. Together with the prior pressure-driven compaction of the CaCO_3_ layer during dead-end precoating, these hydrodynamic conditions favor retention of the sacrificial layer during WWTP-effluent filtration.

**Figure 2:**
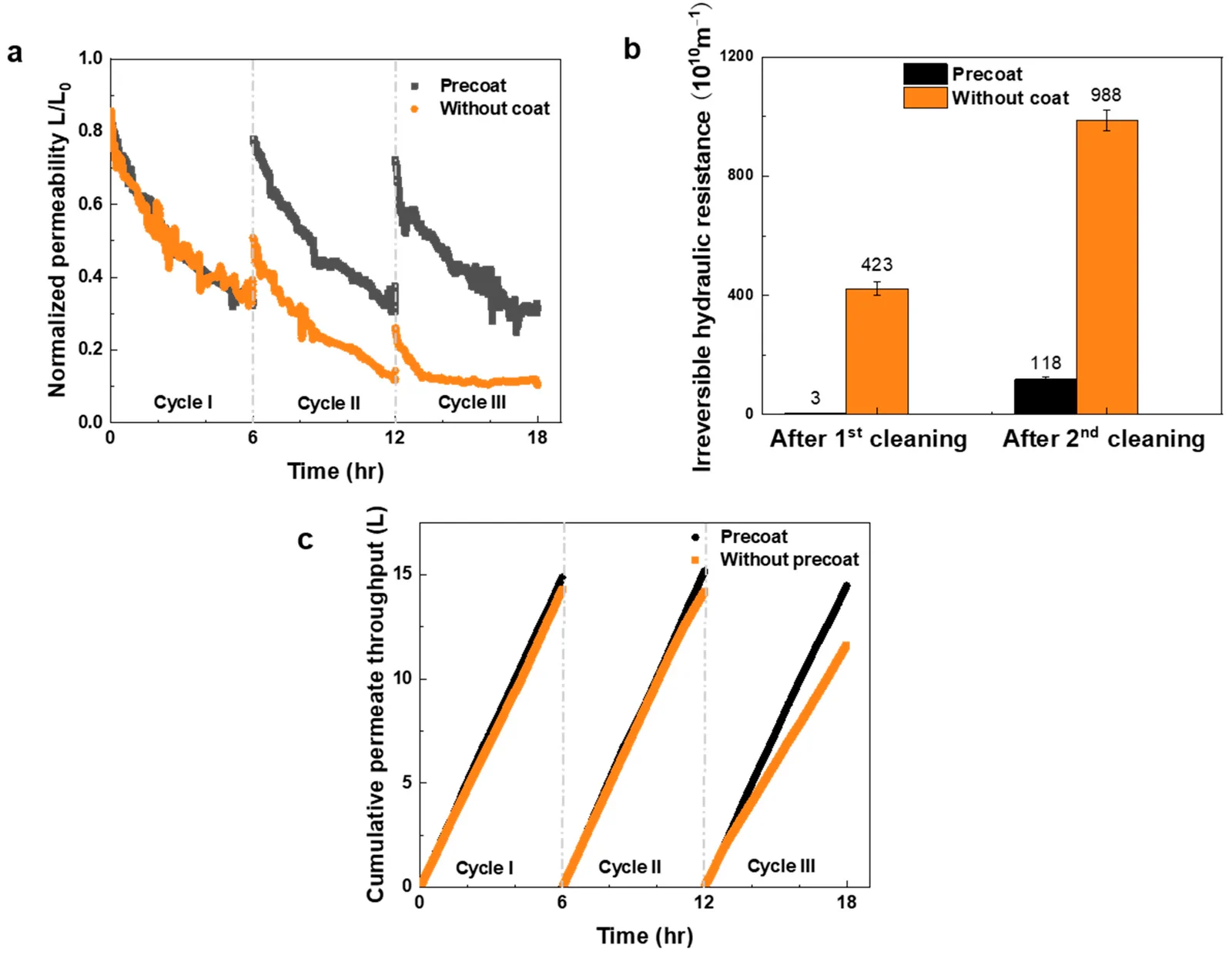
Performance of CaCO_3_-precoated and uncoated ceramic NF membranes over three consecutive WWTP-effluent filtration cycles: (**a**) normalized permeability, (**b**) irreversible hydraulic resistance after cleaning, and (**c**) cumulative permeate throughput.

The membrane used in the filtration experiments had an experimentally determined MWCO of 1021 Da based on PEG retention measurements (Figure S2). This value was used to characterize the membrane employed throughout the present study. The precoated and uncoated ceramic NF membranes were subsequently operated for three consecutive 6-h filtration cycles using WWTP effluent (Figure 2a). During WWTP-effluent filtration, the system was operated at a target permeate flux of 40 L m^−2^ h^−1^, while the cross-flow velocity was maintained at 0.69 m s^−1^. The target flux was maintained by adjusting the TMP within an operating range of approximately 1–10 bar. As fouling accumulated, the hydraulic resistance of the membrane system increased, and a higher TMP was therefore required to sustain the same permeate flux. Accordingly, under constant-flux operation, fouling development is expressed primarily as an increase in the pressure demand required to maintain the preset flux. When the required pressure approached the upper operating limit, the system could no longer fully maintain the target flux, as observed for the uncoated membrane during the later stage of Cycle III. Both systems experienced a progressive decrease in normalized permeability during each filtration cycle, indicating that CaCO_3_ precoating did not prevent foulant accumulation. The main difference between the two systems became apparent after cleaning. Following the first 20 mmol L^−1^ citric acid cleaning step, permeability recovery reached 92% for the precoated membrane but only 34% for the uncoated membrane. After the second cleaning, the precoated membrane still recovered 80% of its initial permeability, whereas the uncoated membrane showed little effective recovery. The permeability recovery values were determined from clean-water permeability measurements immediately after citric-acid cleaning, whereas Figure 2a shows the normalized permeability during subsequent WWTP-effluent filtration. Nevertheless, partial loss or restructuring of loosely attached CaCO_3_ particles during filtration cannot be excluded, and the precoat should therefore be regarded as a stable but sacrificial interfacial layer rather than a permanently immobilized coating.

These results indicate that the principal benefit of the CaCO_3_ precoat was not the complete suppression of fouling during filtration, but the preservation of fouling reversibility during repeated filtration–cleaning cycles. Because both systems were subjected to the same 20 mmol L^−1^ citric acid cleaning procedure for 15 min, the substantially higher permeability recovery is consistent with the beneficial effect of the initial CaCO_3_ precoat and its proposed sacrificial role during acid cleaning. Citric acid forms soluble complexes with Ca^2+^ and promotes dissolution of CaCO_3_-containing deposits [43,44,45]. Dissolution of the initially deposited mineral layer can detach foulants accumulated on or within the precoat, thereby reducing direct association of foulants with the membrane selective layer.

The irreversible hydraulic resistance measurements support this interpretation (Figure 2b). Following the first cleaning step, corresponding to the start of Cycle II, the irreversible resistance was approximately 3 × 10^10^ m^−1^ for the precoated membrane compared with 423 × 10^10^ m^−1^ for the uncoated membrane. Following the second cleaning step, corresponding to the start of Cycle III, the resistance increased to approximately 118 × 10^10^ and 988 × 10^10^ m^−1^, respectively. Although irreversible resistance also accumulated in the precoated system over successive cycles, it remained substantially below that of the uncoated membrane. The markedly higher permeability recovery and lower residual irreversible hydraulic resistance of the precoated membrane indicate that a larger fraction of the accumulated fouling remained removable during repeated filtration–cleaning cycles. Because the measurements at the beginning of Cycles II and III were obtained after cleaning, the reversible resistance generated during the preceding filtration cycle had already been removed and cannot be directly determined at these points. Therefore, the hydraulic data are interpreted as evidence of enhanced fouling reversibility rather than direct proof of the spatial distribution of foulants within the precoat or membrane.

The same difference was reflected in cumulative permeate throughput (Figure 2c). During Cycles I and II, both systems maintained a similar throughput because filtration was operated at a target flux of 40 L m^−2^ h^−1^. During Cycle III, however, the uncoated membrane could no longer maintain the target flux within the imposed 10-bar pressure limit, whereas the precoated membrane sustained substantially greater permeate throughput. Thus, CaCO_3_ precoating and acid-assisted regeneration preserved hydraulic functionality over the three filtration cycles, although progressive fouling remained evident. The hydraulic performance observed here can be placed in the context of previous CaCO_3_-precoating studies. Using a fine-particle CaCO_3_ precoat and a simplified fouling matrix, Li et al. [27] reported permeability recoveries of 93% and 68% following 1st and 2nd cleaning cycles, respectively. In a subsequent study using natural surface water, recoveries of 95% and 87% were obtained for the precoated membrane, compared with 39% and 2% for the uncoated membrane [28]. The corresponding values obtained here with real WWTP effluent, 92% and 80% for the precoated membrane and 34% after the first cleaning for the uncoated membrane, are therefore within the same range, despite the substantially more heterogeneous wastewater matrix. This agreement suggests that the principal benefit of the CaCO_3_ layer, namely preservation of fouling reversibility, is transferable from simplified and surface-water matrices to real WWTP effluent.

### 3.2. Effect of CaCO_3_ Precoating on Solute Removal and Interfacial Mineral Interactions

**Figure 3:**
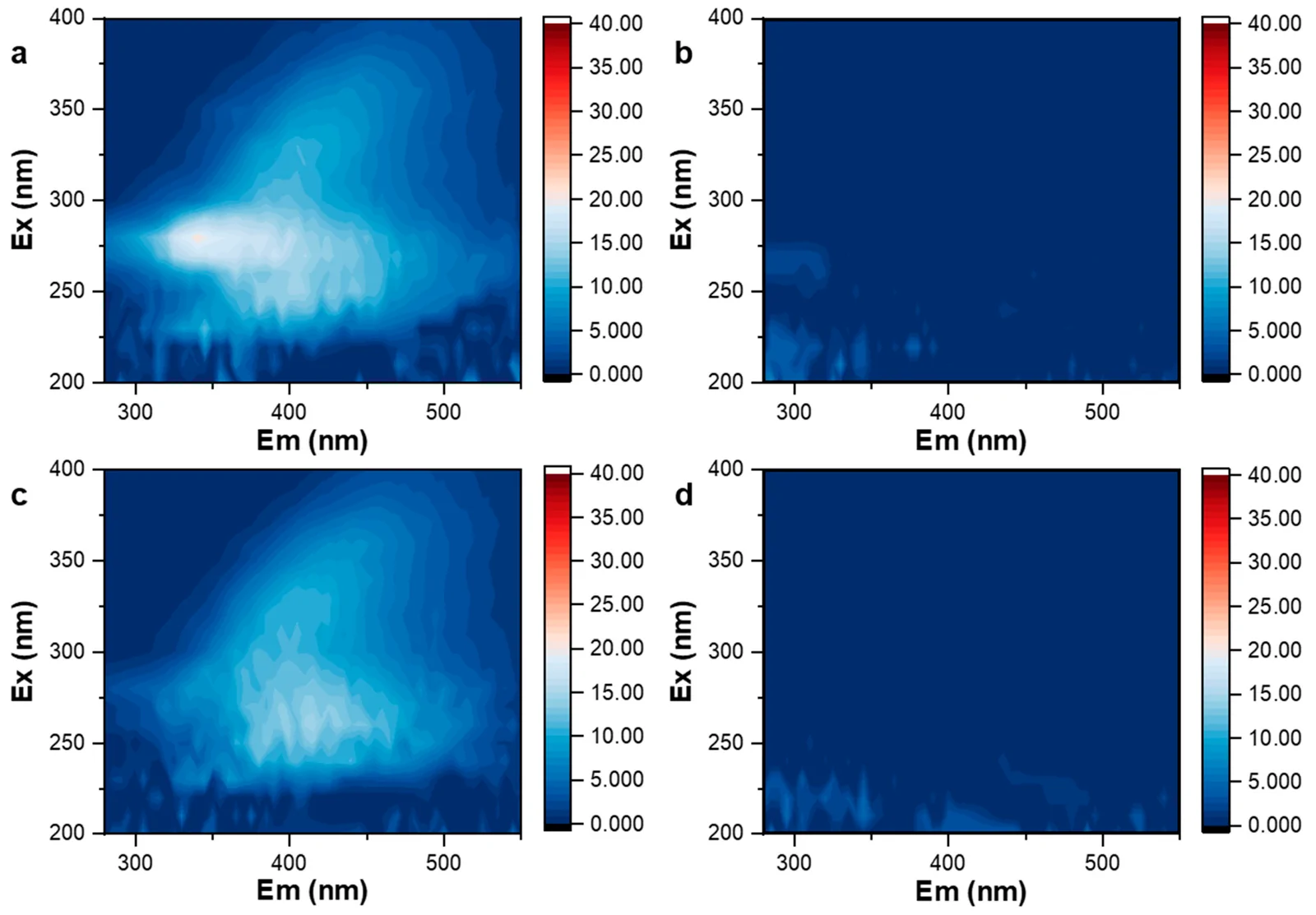
Fluorescence excitation–emission matrix (EEM) spectra of WWTP effluent under different filtration conditions: (**a**) feed before uncoated filtration; (**b**) permeate after uncoated filtration; (**c**) feed before CaCO_3_-precoated filtration; and (**d**) permeate after CaCO_3_-precoated filtration.

EEM fluorescence spectroscopy was used to compare dissolved fluorescent organic matter in the WWTP effluent before and after ceramic NF filtration (Figure 3). The feed samples used for the uncoated and precoated filtration experiments exhibited characteristic protein-like fluorescence in the Ex/Em region of approximately 270–290/300–350 nm and humic-like fluorescence at approximately 230–260/400–500 nm. The corresponding feed-water peak intensities differed between the representative uncoated and precoated experiments, reflecting the natural compositional variability of the real WWTP effluent.

Ceramic NF filtration strongly reduced fluorescence signals in both systems. After filtration with the uncoated membrane, the representative signals at Ex/Em = 280/310 nm and 250/450 nm approached the instrumental detection limit. For the CaCO_3_-precoated membrane, residual intensities at these positions were 0.27 and 0.08, respectively. Overall, no appreciable difference in residual fluorescence was observed between the precoated and uncoated systems. This observation agrees with the DOC measurements, for which apparent removal was 89% for the uncoated membrane and 90% for the precoated membrane (Table 1). Thus, the CaCO_3_ layer substantially affected hydraulic fouling reversibility but produced little additional improvement in bulk DOC removal.

**Figure 4:**
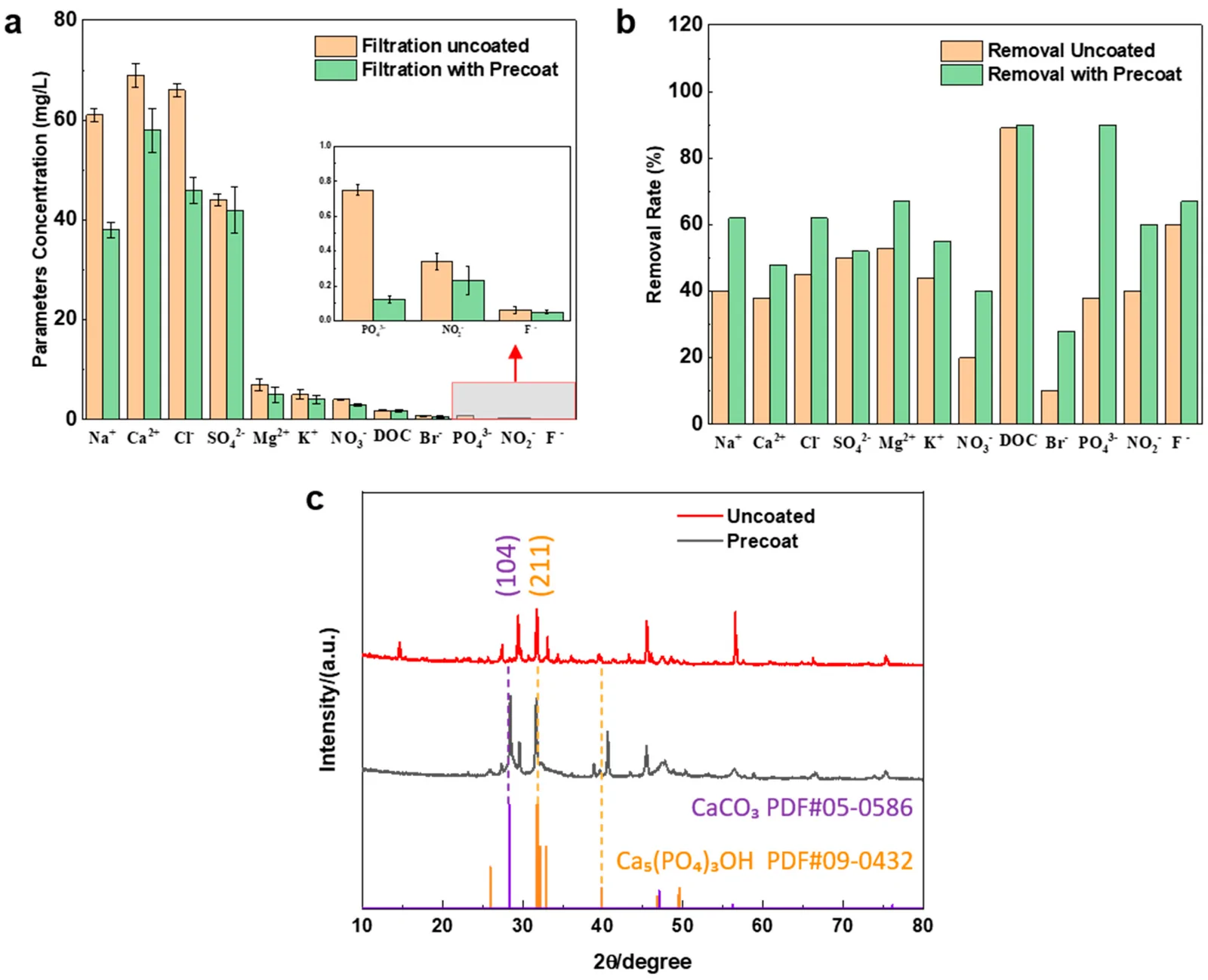
(**a**) Water-quality characteristics of permeates obtained with uncoated and CaCO_3_-precoated ceramic NF membranes; (**b**) apparent removal of selected water-quality constituents; and (**c**) X-ray diffraction (XRD) patterns of uncoated and CaCO_3_-precoated membrane surfaces after WWTP-effluent filtration.

More pronounced differences were observed for several dissolved inorganic constituents (Figure 4 and Table 1). Relative to the uncoated membrane, apparent removal with the CaCO_3_-precoated system increased from 40% to 62% for Na^+^, from 38% to 48% for Ca^2+^, and from 53% to 67% for Mg^2+^. For the anions, apparent removal increased from 45% to 62% for Cl^−^, from 20% to 40% for NO_3_^−^, and from 40% to 60% for NO_2_^−^. Smaller differences were observed for SO_4_^2−^ and F^−^.

The most pronounced change was observed for phosphate. Its concentration decreased from 1.2 mg L^−1^ in the WWTP effluent to 0.75 mg L^−1^ after filtration with the uncoated membrane and to 0.12 mg L^−1^ after filtration with the CaCO_3_-precoated membrane, corresponding to apparent removals of 38% and 90%, respectively. This large increase should not be interpreted solely as enhanced intrinsic membrane rejection. In the precoated system, phosphate can interact directly with the CaCO_3_-rich interfacial layer, and therefore adsorption and mineral precipitation may contribute to its removal in addition to NF separation.

XRD analysis provides supporting evidence for such mineral–solute interactions (Figure 4c). Diffraction features observed for the used precoated membrane overlapped with reference reflections associated with CaCO_3_ and a calcium-phosphate phase. Although the XRD data alone do not establish the quantitative contribution of individual mineral phases, the combination of the XRD response and the strong decrease in dissolved phosphate suggests that the CaCO_3_ precoat acted as a reactive mineral interface in the real WWTP matrix. The phosphate behavior is also consistent with the strong matrix dependence previously reported for ceramic NF. Kramer et al. [3] observed phosphate retention of approximately 76–99% in relatively simple salt solutions as pH increased from 5 to 9, whereas the presence of Mg^2+^ or organic fouling reduced phosphate retention at pH 7 from approximately 92% to 42–43%. The 38% apparent phosphate removal measured here for the uncoated membrane is therefore consistent with the suppression of intrinsic phosphate retention expected in a complex WWTP matrix containing multivalent ions and organic matter. In contrast, the increase to 90% after CaCO_3_ precoating suggests that the mineral layer introduced an additional removal pathway beyond conventional NF rejection, consistent with adsorption and/or Ca–P precipitation indicated by the XRD results.

Although the permeate phosphate concentration decreased to 0.12 mg L^−1^ in the precoated system, this value represents short-term performance under the tested conditions and should not be directly extrapolated to long-term compliance with stringent phosphorus discharge requirements. Because phosphate removal is likely associated partly with adsorption and/or Ca–P mineral formation within the sacrificial CaCO_3_ layer, its reactive capacity is finite and may decrease during prolonged operation. Long-term breakthrough tests, phosphorus mass balances, and repeated precoat-renewal experiments are therefore required to determine the sustainable phosphorus-removal capacity under practical operating conditions.

The effect of CaCO_3_ precoating was ion-dependent, indicating that the mineral layer did not act simply as an additional size-selective barrier. The strongest enhancement was observed for phosphate, for which interaction with the CaCO_3_-rich interface and formation of Ca–P mineral phases likely provided an additional removal pathway. In contrast, SO_4_^2−^ removal increased only marginally from 50% to 52%, suggesting little additional retention by the mineral layer under the present water chemistry. Likewise, F^−^ showed only a moderate increase from 60% to 67%; its relatively low feed concentration and already substantial rejection by the uncoated membrane limited the additional effect of the precoat. These differences indicate that the contribution of the CaCO_3_ layer may depend on ion-specific mineral–solute interactions. The improved apparent removal of several ionic species may similarly reflect a combination of membrane separation and interactions within the mineral precoat. Because the present experiments did not independently quantify membrane surface charge, adsorption capacity, or mineral precipitation for each ion, these contributions cannot be separated quantitatively. Accordingly, the observed concentration reductions are referred to here as apparent removals rather than intrinsic membrane rejection values.

Taken together, the results show two distinct functions of the CaCO_3_ precoat. Its primary benefit was hydraulic: it preserved fouling reversibility during repeated operation and cleaning. At the same time, the precoat introduced an additional mineral interface capable of interacting with selected dissolved constituents, most notably phosphate. In contrast, DOC removal remained essentially unchanged, indicating that the hydraulic protection provided by the precoat did not compromise the bulk organic-matter separation of the ceramic NF membrane.

### 3.3. Comparison with Previous Studies and Practical Implications

Taken together, comparison with previous CaCO_3_-precoating studies reveals both consistency and an important matrix effect. Permeability recovery remained comparable to that achieved with simplified and natural surface-water matrices, demonstrating that the sacrificial-layer concept remains effective in real WWTP effluent. In contrast, the progressive accumulation of irreversible resistance and the strongly ion-dependent removal behavior indicate that the complexity of WWTP effluent imposes a considerably greater challenge than the simpler water matrices evaluated previously. The precoat should therefore not be viewed as a universal antifouling barrier or as an additional NF selective layer. Its main function is to maintain a greater fraction of accumulated fouling in a removable form while providing a reactive mineral interface for selected solutes.

This distinction is important for practical application. Previous work demonstrated that a single CaCO_3_ precoat could support multiple filtration–cleaning cycles under controlled conditions [28], whereas the present study demonstrates persistence over three cycles in real WWTP effluent. However, the continued increase in irreversible resistance from Cycle II to Cycle III indicates that the protective capacity is finite. Consequently, practical operation will require optimization of the trigger for precoat renewal, rather than assuming that one coating can provide indefinite protection. The present three-cycle experiments should therefore be regarded as evidence of transferability to a complex wastewater matrix rather than as a demonstration of long-term operational lifetime.

## 4. Conclusions

This study evaluated ceramic NF membranes coated with CaCO_3_ for the filtration of WWTP effluent. The main conclusions are as follows:

• CaCO_3_ precoating imposed only a small initial hydraulic penalty but substantially preserved permeability recovery during repeated filtration and cleaning cycles. Recovery reached 92% after the first cleaning and 80% after the second cleaning, compared with only 34% for the uncoated membrane after the first cleaning.
• The substantially lower irreversible hydraulic resistance of the precoated membrane indicates that the major benefit of the sacrificial layer was to maintain fouling in a more removable form rather than to completely prevent foulant accumulation.
• DOC removal remained essentially unchanged, whereas apparent phosphate removal increased from 38% to 90%. XRD features are consistent with CaCO_3_, and a Ca–P mineral phase suggests that mineral and solute interactions within the precoat may have contributed to phosphate removal.

## Figures and Tables

**Table 1:** Water-quality characteristics and apparent removal by uncoated and CaCO_3_-precoated ceramic NF membranes.

	Units	WWTP Effluent	Uncoated Permeate	Precoated Permeate	Removal Uncoated	Removal Precoated
pH	-	8.5	8.2	8.1	-	-
Conductivity	μS cm^−1^	1790	1023	756	43%	58%
DOC	mg L^−1^	17	1.84	1.7	89%	90%
Na^+^	mg L^−1^	101	61	38	40%	62%
K^+^	mg L^−1^	9	5	4	44%	55%
Mg^2+^	mg L^−1^	15	7	5	53%	67%
Ca^2+^	mg L^−1^	111	69	58	38%	48%
F^−^	mg L^−1^	0.15	0.06	0.05	60%	67%
Cl^−^	mg L^−1^	121	66	46	45%	62%
NO_3_^−^	mg L^−1^	5	4	3	20%	40%
Br^−^	mg L^−1^	0.83	0.71	0.60	10%	28%
NO_2_^−^	mg L^−1^	0.57	0.34	0.23	40%	60%
PO_4_^3−^	mg L^−1^	1.2	0.75	0.12	38%	90%
SO_4_^2−^	mg L^−1^	88	44	42	50%	52%

## Data Availability

The data presented in this study are available from the corresponding author upon reasonable request.

## Supplementary Materials

The following supporting information can be downloaded at https://www.mdpi.com/article/10.3390/membranes16090306/s1.
