# Supplementary material for: Sacrificial CaCO3 Precoating Preserves Fouling Reversibility of Ceramic Nanofiltration During Filtration of Real Wastewater Treatment Plant Effluent
Source: Membranes (Basel). 2026 Sep 18;16(9):306. doi: 10.3390/membranes16090306 (PMC13609071; doi:10.3390/membranes16090306)
Supplement: Supplementary file 1 [file membranes-16-00306-s001.zip › membranes-4577378-supplementary.pdf]

membranes

Article

Supplementary Information: Sacrificial $CaCO_3$ Precoating Preserves Fouling Reversibility of Ceramic Nanofiltration During Filtration of Real Wastewater Treatment Plant Effluent

Yuke Li *, Luuk C. Rietveld and Sebastiaan G.J. Heijman

Section of Sanitary Engineering, Department of Water Management, Faculty of Civil Engineering and Geosciences, Delft University of Technology, Stevinweg 1, 2628 CN Delft, The Netherlands

***Correspondence: Yuke Li, Section of Sanitary Engineering, Department of Water Management, Faculty of Civil Engineering and Geosciences, Delft University of Technology, Stevinweg 1, 2628 CN Delft, The Netherlands; Tel: +86-18763899375; yukeli94@163.com, L.Li-6@tudelft.nl**

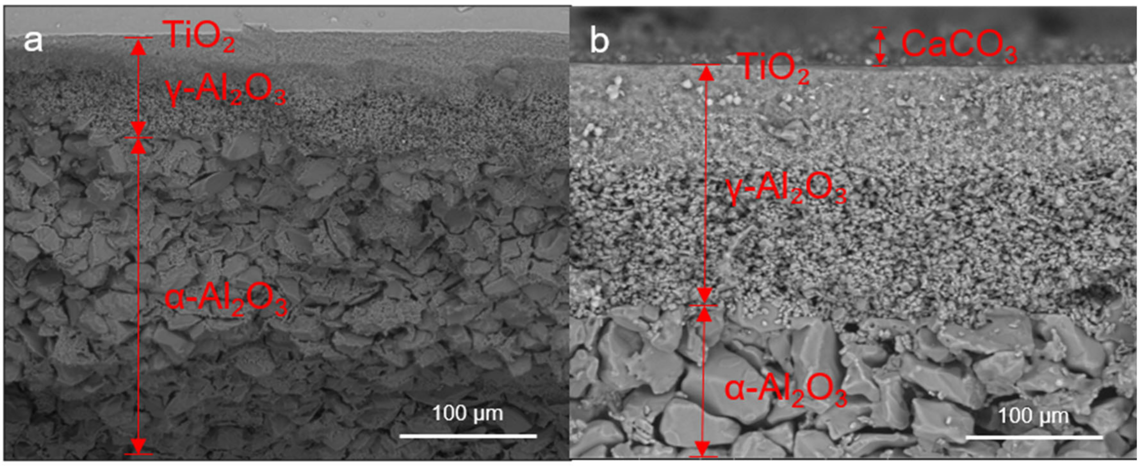

Figure S1. Cross-sectional SEM images of the ceramic NF membrane before and after $CaCO_3$ precoating: (a) pristine membrane showing the α-Al_2O_3 support, γ-Al_2O_3 intermediate layer, and TiO_2 selective layer; (b) $CaCO_3$-precoated membrane showing the additional $CaCO_3$ layer deposited on the TiO_2 selective surface. Adapted from Ref. [1] under the terms of the Creative Commons CC BY 4.0 license.

membranes

MDPI

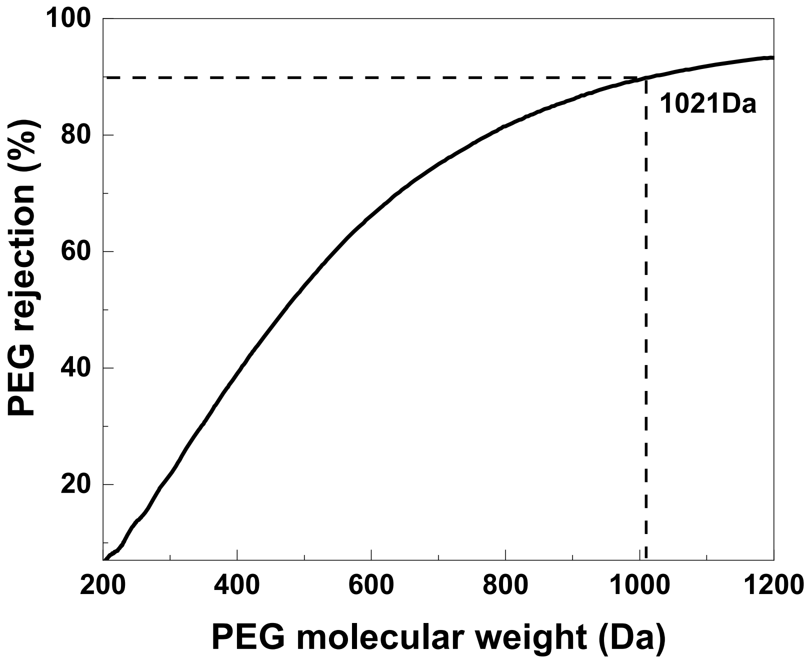

Figure S2. Molecular weight cut-off (MWCO) characterization of the ceramic nanofiltration membrane based on PEG retention.

Table S1. Water-quality characteristics of WWTP effluent and permeates obtained with uncoated and $CaCO_3$-precoated ceramic NF membranes. Values are reported as mean ± standard deviation (n = 3).

	Units	WWTP effluent	Uncoated permeate	Precoated permeate
pH	-	8.5±0.2	8.2±0.4	8.1±0.2
Conductivity	μS cm^{-1}	1790±46	1023±22	756±31
DOC	mg L^{-1}	17±0.21	1.84±0.09	1.72±0.11
Na^+	mg L^{-1}	100.53±1.62	60.98±1.31	38.39±1.24
K^+	mg L^{-1}	9.11±0.79	5.40±0.76	3.64±0.84
Mg^{2+}	mg L^{-1}	14.78±1.48	7.01±1.15	5.03±1.23
Ca^{2+}	mg L^{-1}	110.63±4.37	68.99±2.36	57.57±2.49
F^-	mg L^{-1}	0.15±0.01	0.06±0.02	0.05±0.001
Cl^-	mg L^{-1}	120.55±2.61	65.68±1.44	45.74±1.37
NO_3^-	mg L^{-1}	5.35±0.22	4.32±0.14	2.74±0.20
Br^-	mg L^{-1}	0.83±0.21	0.71±0.17	0.60±0.35
NO_2^-	mg L^{-1}	0.57±0.08	0.34±0.05	0.23±0.04
PO_4^{3-}	mg L^{-1}	1.2±0.11	0.75±0.03	0.12±0.002
SO_4^{2-}	mg L^{-1}	88.07±4.64	44.26±1.23	41.58±1.15

Reference

1. Li, Y., L.C. Rietveld, and S.G.J. Heijman, *Analysis of fouling control strategies in ceramic nanofiltration membranes: $CaCO_3$ precoating versus continuous dosing.* Desalination, (2026). **637**:120502 https://doi.org/10.1016/j.desal.2026.120502.
